# The 2b protein and C-terminal region of the 2a protein indispensably facilitate systemic movement of cucumber mosaic virus in radish with supplementary function by either the 3a or the coat protein

**DOI:** 10.1186/s12985-020-01303-3

**Published:** 2020-04-07

**Authors:** Yu Yu Khaing, Yudai Kobayashi, Minoru Takeshita

**Affiliations:** Laboratory of Plant Pathology, Faculty of Agriculture, Department of Agricultural and Environmental Sciences, University of Miyazaki, Gakuenkibanadainishi 1-1, Miyazaki, 889-2192 Japan

**Keywords:** Cucumber mosaic virus, Host-specificity, Systemic movement, Radish

## Abstract

**Background:**

In *Raphanus sativus* (Japanese radish), strain D8 of cucumber mosaic virus (CMV-D8) establishes a systemic infection and induces mild mosaic on upper, non-inoculated leaves, whereas strain Y of CMV (CMV-Y) causes only a local infection in the inoculated leaves. Here, we further analyzed the specific viral factor(s) of CMV-D8 that is (are) indispensable for systemic infection in Japanese radish.

**Methods:**

To identify which genomic RNA(s) is (are) involved in systemic infection in radish, we carried out a pseudorecombination analysis between CMV-D8 and CMV-Y. With recombination analyses between CMV-D8 and CMV-Y using mutant/recombinant RNA2s, chimeric and point-mutated RNA3s, we identified viral factors that are indispensable for systemic infection.

**Results:**

Viral RNA2 and RNA3 of CMV-D8 facilitated efficient virus spread into the upper, non-inoculated plant tissues of radish (cv. Tokinashi), but not those of CMV-Y. Recombinant RNA2s demonstrated that the 2b protein (2b) and the C-terminus of the 2a protein (2a) of CMV-D8 have a crucial role in systemic infection. In addition, we used chimeric and point-mutated RNA3s to that Pro^17^ and Pro^129^ in the coat protein (CP) of CMV-D8 are involved in efficient systemic infection and that Ser^51^ in the 3a protein (3a) of CMV-D8 has positive effects on systemic spread. The results suggested that these viral factors facilitate systemic infection of CMV-D8 in Japanese radish.

**Conclusion:**

The C-terminal region of 2a, the entire region of 2b, and supplementary function of either Ser^51^ in 3a or Pro^17^/Pro ^129^ in CP confer systemic infectivity on CMV-D8 in radish. These results further elucidate the complex interaction of viral proteins of CMV to complete systemic infection as a host-specific manner.

## Background

Infecting more than 1200 dicotyledonous and monocotyledonous plant species, cucumber mosaic virus (CMV; genus *Cucumovirus*, family *Bromoviridae*) is one of the most important viruses worldwide [10, 27, 30]. CMV induces various kinds of symptoms, which depend on the host plant species and virus strain, ranging from little or no visible symptoms to severe mosaic associated with stunting and leaf deformations [2, 4, 12, 15, 20, 23, 28].

The numerous strains reported for CMV have been divided into two subgroups I (IA, IB) and II based on serological relationships and nucleotide sequence homology [29, 30]. CMV contains three positive-sense RNA segments designated RNA1, RNA2 and RNA3 [10, 30]. RNA1 and RNA2 encode protein 1a and 2a, respectively, and are involved in viral RNA replication [29]. Protein 2b, encoded by subgenomic RNA4A from RNA2 is well known as a viral silencing suppressor [21, 24] and is also responsible for long-distance viral movement [6, 42] and determining pathogenicity and symptom types [7]. The movement protein (3a), essential for cell-to-cell virus movement [18], and the coat protein (CP) are encoded by RNA3. Choi et al. [4] reported that 2a and 3a of CMV independently affect virus spread and symptom induction in squash. The CP is also critical for cell-to-cell and systemic movement of the virus, determining pathogenicity, virus accumulation, symptom induction, and vector transmission [19, 23, 26, 29, 33, 43]. Cell-to-cell and long-distance movement of viruses are the most important factor for systemic infection in plants [3].

In Japan, radish is the most widely grown root vegetable, and systemic mosaic is common [41, 44]. Severe mosaic disease is mostly associated with a mixed infection with turnip mosaic virus and CMV [34]. Adhab and Alani [1] reported that mechanical inoculation with a crude sap from CMV-symptomatic radish leaves caused a systemic mild mosaic on radish (*Raphanus sativus*). Takeshita et al. [39] reported that CMV-Y in subgroup IA causes only a local infection in the inoculated leaves of radish (cv. Akidumari), whereas CMV-D8, which is a natural pseudorecombinant strain containing RNA1 and RNA2 from subgroup IA and RNA3 from subgroup IB induces mild systemic mosaic (Additional file 1: Figure S1A), and its RNA2 and RNA3 determine the pathogenicity in radish. However, which regions or viral factors in RNA2 and RNA3 are responsible for systemic infection in the plants has remained unclear. Therefore, here we focused on further elucidating the genetic information in RNA2 and RNA3 of CMV-D8 that confers its ability to induce systemic infection in Japanese radish.

## Methods

### Plants, viruses

Radish (*Raphanus sativus* L. cv. Tokinashi) was grown in a growth chamber at 25 °C for use as test plants. Strains CMV-Y and CMV-D8 and their maintenance in *Nicotiana benthamiana* plants were previously described by Takeshita et al. [39].

### Construction of cDNA clones of CMV-Y, CMV-D8, recombinant and mutant RNA2s, chimeric and point-mutated RNA3s

cDNA clones of CMV-Y RNAs and CMV-D8 RNAs were prepared by Suzuki et al. [37] and Takeshita et al. [39], respectively. Pseudorecombinant combinations were prepared by exchanging the tripartite genomes between CMV-D8 and CMV-Y using in vitro transcripts derived from the cDNA clones. PCR-based site-directed mutagenesis using oligonucleotide primers was performed to construct recombinant/mutant RNA2s and point-mutated RNA3s according to the methods of Takeshita et al. [40]. Chimeric RNA3s were created by reciprocal exchange using a BamHI site upstream of the T7 promoter and an internal SalI site of the each full-length RNA3 cDNA clone between two isolates as described by Takeshita et al. [40]. The primer sequences for point-mutated RNA3s and recombinant/mutant RNA2 plasmid constructions are listed in Additional file 2: Table S1 and Additional file 3: Table S2, respectively.

### In vitro transcription

Capped transcripts from NotI-linearized full-length cDNA clones of CMV RNAs, recombinant/mutant RNA2s, chimeric and point-mutated RNA3s were obtained by in vitro transcription reactions with bacteriophage T7 RNA polymerase in the presence of m7GpppG essentially as described by Suzuki et al. [37]. For example, for point mutation derivatives, Y3MP51NS indicates point-mutated Y3 in which asparagine was replaced by serine at position 51 in the MP gene; Y3CP17LP indicates point-mutated Y3 in which leucine was replaced by proline at position 17 in the CP gene and Y3CP129SP indicates point-mutated Y3 in which serine was replaced by proline at position 129 in the CP gene. The transcripts were combined in equal amounts to prepare various reassortants, and then used to mechanically inoculate *N. benthamiana* plants for further use. The schematic diagram of recombinant/mutant RNA2s, chimeric and point-mutated RNA3s used in this study are shown in Additional file 4: Figures S2 and S3. The mutated sequences were confirmed by nucleotide sequence analysis.

### Inoculation

Systemically infected leaves from *N. benthamiana* plants were ground in 0.1 M phosphate buffer, pH 7.0 with a pestle. Then, cotyledons of 7-day-old radish seedlings were dusted with carborundum powder and mechanically inoculated with infected crude sap from *N. benthamiana* plants, which were already challenged with each CMV wild type and each mutant separately. Cotyledons were inoculated only with buffer as mock-inoculated plants. Eight individual plants were used for each treatment in the inoculation assays, which were done two times.

### Tissue immunoblotting assay

Accumulation and spread of virus in the plants was assayed using tissue print immunoblots as described by Lin et al. [22] with some modifications. Inoculated cotyledons at 10 days post inoculation (dpi) and upper, non-inoculated leaves of radish plants at 21 dpi were detached, rolled into a tight core, and cut with a new razor blade for each sample. Root tissues of the plants at 21 dpi were also detected to assess the systemic spread of virus. The freshly cut surface was pressed onto a nitrocellulose membrane (0.45 μm, TOYO, Japan) to obtain a tissue print. The membranes were air-dried, then incubated with shaking in a blocking solution of sodium phosphate buffer pH 7.2, 0.14 M NaCL, 0.1% Tween 20 (v/v) (PBST), 5% skimmed milk (v/v) and 1% TritonX 100 (v/v) for at least 1–3 h until the green color disappeared. Then, the membranes were reacted with antiserum against CMV-Y CP diluted in PBST containing 0.3% bovine serum albumin (PBST-BSA) at room temperature for 2 h. Unbound antibody was removed by washing with PBST, and the membranes were incubated in PBST-BSA containing goat anti-rabbit IgG conjugated to alkaline phosphate for 1 h, then rinsed again in PBST. Virus was detected by a colorimetric reaction using nitroblue tetrazolium and 5-bromo-4-chloro-3-indolyl-phosphate.

### RNA extraction and reverse transcription polymerase chain reaction (RT-PCR)

Total RNAs were extracted from the inoculated cotyledons (10 dpi) and upper non-inoculated leaves (21 dpi) of the plants using RNAiso Plus (TaKaRa, Japan) according to the manufacturer’s instructions. The quality and quantity of the extracted RNAs was assessed using NanoDrop 2000 Spectrophotometer (Thermo Scientific) and stored at − 80 °C until used. The first strand of cDNA was synthesized with ReverTra Ace (TOYOBO, Japan) and was subjected to RNA3-specific PCR using KOD FX Neo (TOYOBO, Japan) and thermocycling at 94 °C for 2 min; 40 cycles at 98 °C for 10 s, 58 °C for 30 s, and 68 °C for 2 min. Amplified RT-PCR products were separated in 2% agarose gel and stained with ethidium bromide. The expected product size was 2.2 kb. *ACTIN2/7* was used as the control. The primers used in this step are shown in Additional file 5: Table S3.

### Relative quantitative reverse transcription polymerase chain reaction (RT-qPCR)

cDNAs for quantitative RT-PCR analysis were prepared using ReverTra Ace qPCR 5x RT Master Mix II with gDNA Remover (TOYOBO, Japan) and the manufacturer’s instructions in 20 μl of total reaction volume. Primers used for quantitative RT-PCR amplification of RNA2 were selected to anneal to corresponding identical sequences between CMV-Y and CMV-D8. *ACTIN2/7* and glyceraldehyde 3-phosphate dehydrogenase (*GAPDH*) genes [9] were used as reference genes for relative quantification (Additional file 5: Table S3).

Relative RT-qPCR was performed with a Thermal Cycler Dice Real Time System II (TaKaRa, Japan) and 96-well plates. Total reaction volumes of 20 μl contained THUNDERBIRD SYBR qPCR Mix (10 μl), 7 μl of nuclerase free water, 1 μl each of the forward and reverse primer pairs (10 pmol) and 1 μl of cDNA template. Each cDNA sample from three biological replicates was amplified in triplicate in a 96-well optical plate. The protocol was 95 °C for 1 min; 40 cycles of 10 s at 95 °C, 30 s at 60 °C and 20 s at 72 °C according to the manufacturer’s instruction; and a final standard dissociation analysis was run for 10 s at 95 °C, 5 s at 60 °C and 10 s at 95 °C to obtain the melting profiles. Levels of CMV RNA2 were analyzed by Multiplate RQ software (TaKaRa, Japan) using the comparative cycle threshold (Ct) (∆∆Ct) method. The significance of any differences in means of viral RNA levels was analyzed using Tukey-Kramer test at *P* < 0.05 or *P* < 0.01.

## Results

### CMV-D8 systemically spread in radish

**Fig. 1:**
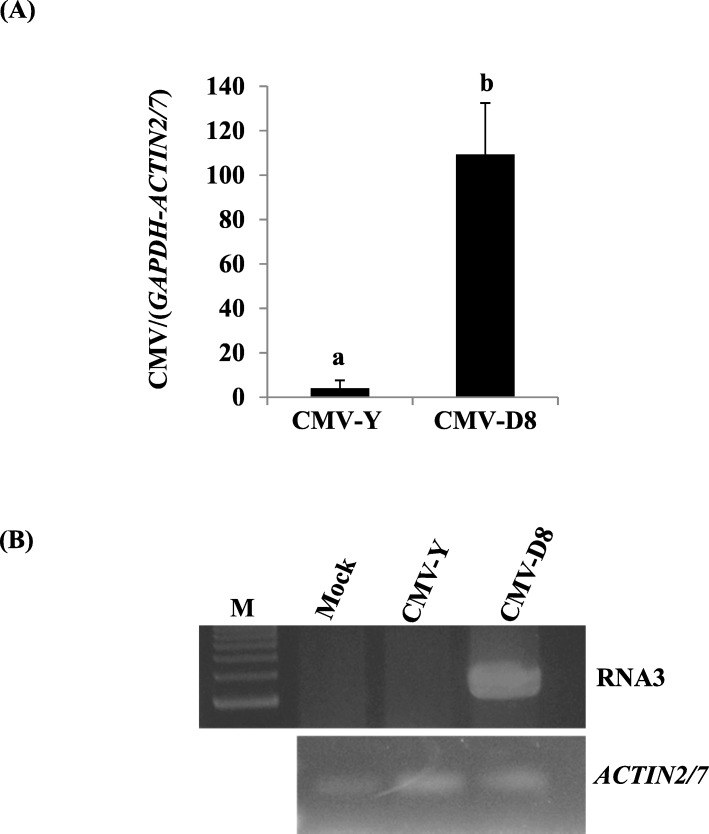
Accumulation of cucumber mosaic virus (CMV) in radish (cv. Tokinashi). **a** Relative accumulation levels of CMV RNA2 in inoculated cotyledons were quantified by relative RT-qPCR. **b** Detection of CMV in the upper, non-inoculated leaves by RNA3-targeted RT-PCR. Data are mean values from three technical replicates with three biological replicates for each cDNA; vertical bars indicate standard errors. Data were analyzed by the ∆∆Ct method followed by analysis of significant differences using Tukey test (*P* < 0.05 or *P* < 0.01). Different letters on the bars indicate statistically significant difference between the leaf samples infected with CMV-Y and CMV-D8. Data were first normalized using the *GAPDH*/ *ACTIN2/7* genes

In the initial step of this study, we examined the pathogenicity of CMV-D8 and CMV-Y using radish cv. Tokinashi because cv. Akidumari used by Takeshita et al. [39] is no longer commercially available. Relative levels of CMV-D8 and CMV-Y in the plants at 10 days post inoculation (dpi) and 21 dpi was compared in total RNAs extracted from the inoculated and the upper, non-inoculated leaves, respectively, using relative RT-qPCR targeting CMV RNA2. In the cotyledons, the viral level in CMV-D8-inoculated plants was significantly higher than in plants inoculated with CMV-Y (Fig. 1a). CMV-D8 accumulated in the upper, non-inoculated leaves, whereas CMV-Y was not detected by RT-PCR targeting RNA3 (Fig. 1b), suggesting that the pathogenicity of CMV-D8 and CMV-Y showed a similar outcome in the two radish cultivars (Akidumari and Tokinashi).

### Genetic information in CMV-D8 RNAs 2 and 3 involved in systemic infection of radish

**Fig. 2:**
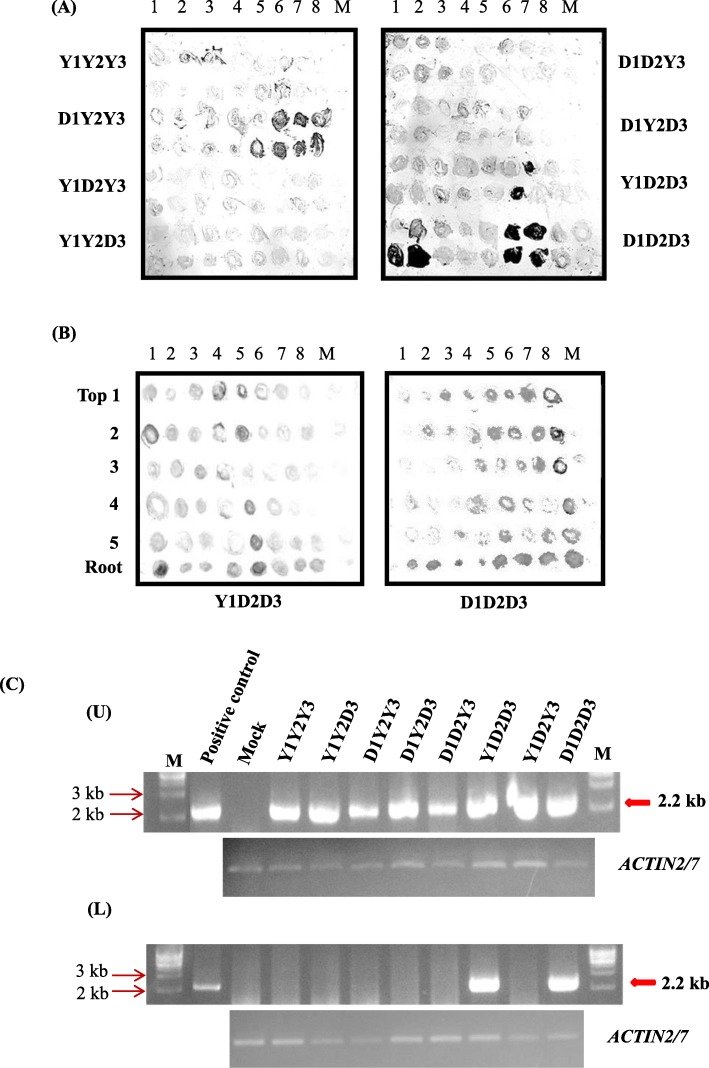
Accumulation of CMV in radish (cv. Tokinashi). **a** Detection of CMV in inoculated cotyledons at 10 dpi and **b** in the upper, non-inoculated leaves and roots at 21 dpi by tissue immunoblotting using antiserum against CMV-Y CP. Cotyledons of 7-day-old seedlings were mechanically inoculated with CMV-Y, CMV-D8, and their pseudorecombinants. Numbers at the top represent individual test plants, and numbers on the left indicate leaf positions from the top of the plants. M, mock-inoculated plants. **c** Results of RT-PCR targeting RNA3 using total RNAs from inoculated cotyledons (U, upper row) and upper, non-inoculated leaves (L, lower row) of radish

To determine which genomic RNA(s) is (are) associated with systemic movement of CMV, pathogenicity of pseudorecombinants between CMV-D8 and CMV-Y was examined in radish (cultivar Tokinashi). On the leaves used for immunotissue blots to detect the virus in the inoculated plants, neither CMV-Y nor CMV-D8 had induced any visible symptoms in the upper leaves of radish by 21 dpi (Additional file 1: Figure S1B). Viral accumulation was detected in cotyledons at 10 dpi and in upper, non-inoculated leaves at 21 dpi. As shown in Table 1, all of the pseudorecombinants and wild-type strains infected the cotyledons. In contrast, only pseudorecombinant Y1D2D3 and CMV-D8 spread to the upper, non-inoculated leaves of radish. The results in Fig. 2a revealed that CMV accumulated in all of the cotyledons of the tested plants inoculated with CMV-Y, CMV-D8, and their pseudorecombinants. However, Y1D2D3 and CMV-D8 only spread to the upper leaves and roots of the plants (Fig. 2b). The other pseudorecombinants such as D1D2Y3, D1Y2D3, Y1Y2D3, Y1D3Y3, and D1Y2Y3, and CMV-Y did not move systemically in the plants, indicating that both CMV-D8 RNA2 and RNA3 were needed for systemic infection of the plants. The results for the RT-PCR targeting RNA3 shown in Fig. 2c revealed that viral RNA was detected in the cotyledons of the plants inoculated with the wild types and with the pseudorecombinants, but only pseudorecombinant Y1D2D3 and CMV-D8 were detected in the upper leaves of the plants. The results were the same as those reported by Takeshita et al. [39] for cv. Akidumari.

**Table 1:** Pseudorecombination analysis in radish by tissue immuno-blotting assay

Inoculum	Inoculated leaves^a^	Upper leaves^b^
Mock	0/8^c^	0/8^c^
D1D2D3(CMV-D8)	8/8	8/8
Y1Y2Y3(CMV-Y)	8/8	0/8
Y1Y2D3	8/8	0/8
D1Y2Y3	8/8	0/8
D1Y2D3	8/8	0/8
D1D2Y3	8/8	0/8
Y1D2D3	8/8	8/8
Y1D2Y3	8/8	0/8

Data were recorded at ^a^10 dpi; ^b^ 21 dpi; ^c^Number of infected plants/number of inoculated plants. Some samples with very low levels of viral accumulation were included as positive

### Entire region of 2b and C-terminal region of 2a from CMV-D8 cooperatively play a crucial role in systemic movement

**Fig. 3:**
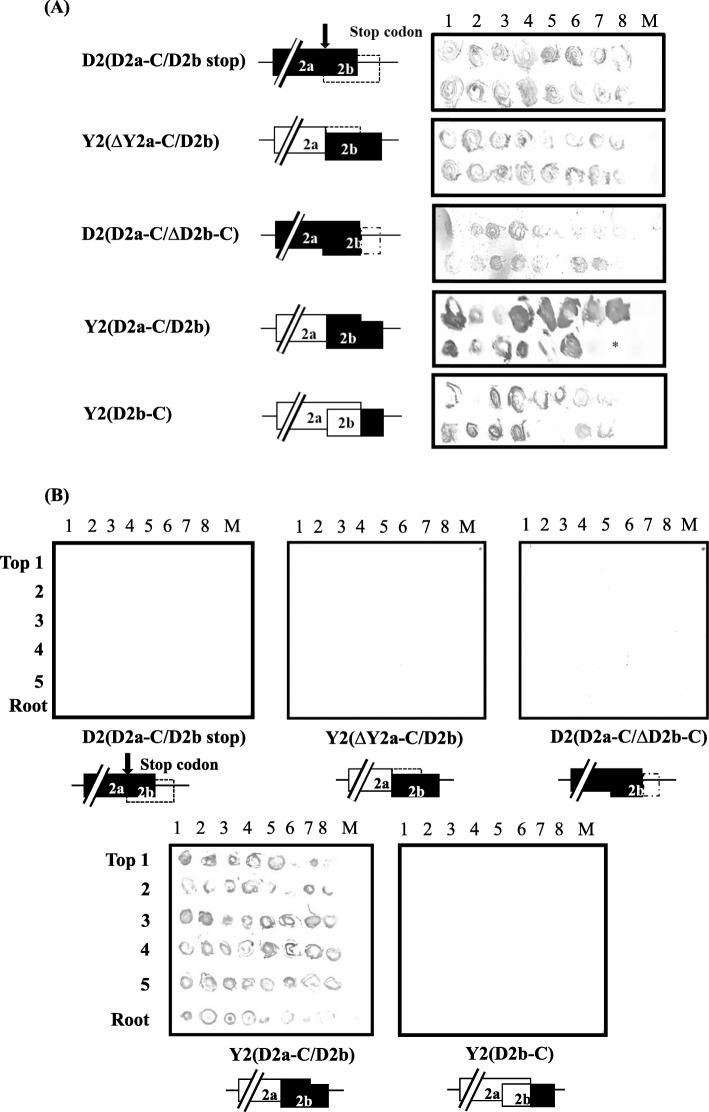
Accumulation of CMV in radish (cv. Tokinashi). **a** Detection of CMV in inoculated cotyledons at 10 dpi and **b** in upper, non-inoculated leaves and roots at 21 dpi by tissue immunoblotting using antiserum against CMV-Y CP. Cotyledons of 7-day-old seedlings were mechanically inoculated with RNA2 mutants, combined with Y RNA1 and D8 RNA3. Numbers at top represent individual test plants; numbers on left indicate leaf positions from the top of the plants. M, mock-inoculated plants. Asterisks indicate dead or senesced leaves that could not be analyzed

**Fig. 4:**
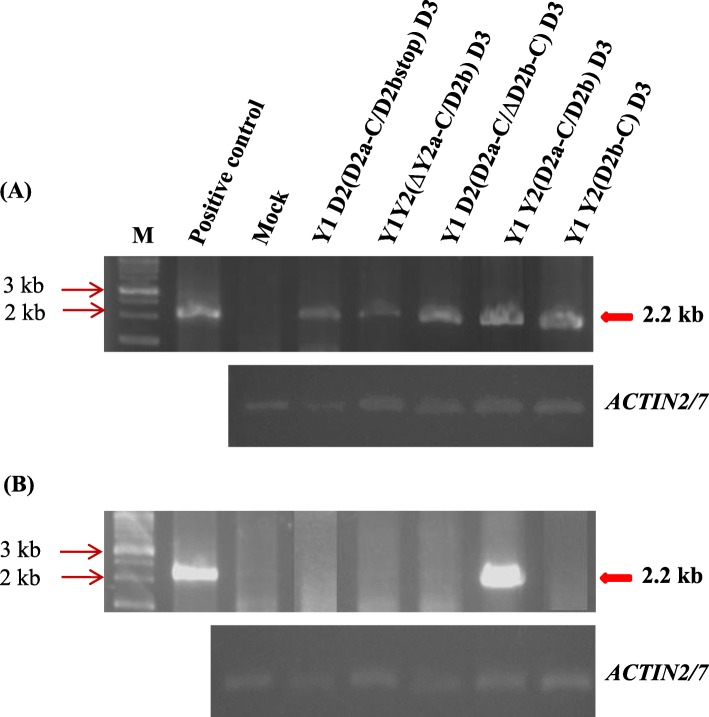
**a** Detection of CMV in inoculated cotyledons at 10 dpi and **b** in upper, non-inoculated leaves at 21 dpi by RT-PCR targeting RNA3. Total RNAs were extracted from plants inoculated with RNA2 mutants, combined with Y RNA1 and D8 RNA3

To elucidate which viral protein(s) is (are) involved in the systemic infectivity of CMV-D8 in radish, we first focused on the 2a and 2b genes encoded by RNA2. Several recombinants associated with both viral genes, designated D2(D2a-C/D2b stop), Y2(∆Y2a-C/D2b), D2(D2a-C/∆D2b-C), Y2(D2a-C/D2b) and Y2(D2b-C) were constructed using infectious clones of RNA2 from CMV-D8 and CMV-Y (Additional file 4: Figure S2). The alignment of the amino acid sequences of the 2a and 2b proteins between CMV-D8 and CMV-Y is shown in Additional file 6: Figure S4. Systemic infectivity of the CMV mutants (mutated RNA2s, Y RNA1 and D8 RNA3) was first evaluated by tissue immunoblotting, which showed that all of the inoculated cotyledons were infected. However, the accumulation level of Y2(D2a-C/D2b), which includes the C-terminal region of 2a ORF and the entire 2b ORF from CMV-D8, was somewhat higher than for the other mutants (Table 2, Fig. 3a). Virus only accumulated in the upper, non-inoculated leaves in plants inoculated with Y2(D2a-C/D2b) (Fig. 3b). RT-PCR of RNA3 showed that CMV was present in all of the inoculated cotyledons; however, only Y1Y2(D2a-C/D2b)D3 was detected in the upper leaves of the plants (Fig. 4). The results suggested that the C-terminal region of the 2a ORF, which overlaps two-thirds of the region from N-terminus of 2b ORF, and the entire 2b ORF are responsible for systemic infection of CMV-D8 in radish.

**Table 2:** Mutant and recombinant RNA2s analysis in radish by tissue immuno-blotting assay

Inoculum	Inoculated leaves^a^	Upper leaves^b^
Mock	0/8^c^	0/8^c^
Y1 D2(D2a-C/D2bstop) D3	8/8	0/8
Y1 Y2(∆Y2a-C/D2b) D3	8/8	0/8
Y1 D2(D2a-C/∆D2b-C) D3	8/8	0/8
Y1 Y2(D2a-C/D2b) D3	8/8	8/8
Y1 Y2(D2b-C) D3	8/8	0/8

Data were recorded at ^a^10 dpi; ^b^21 dpi; ^c^Number of infected plants/number of inoculated plants. Some samples with very low levels of viral accumulation were included as positive

### Either amino acid 51 in the MP or 17/129 in the CP supplementally facilitate systemic infection of CMV-D8 in radish

**Fig. 5:**
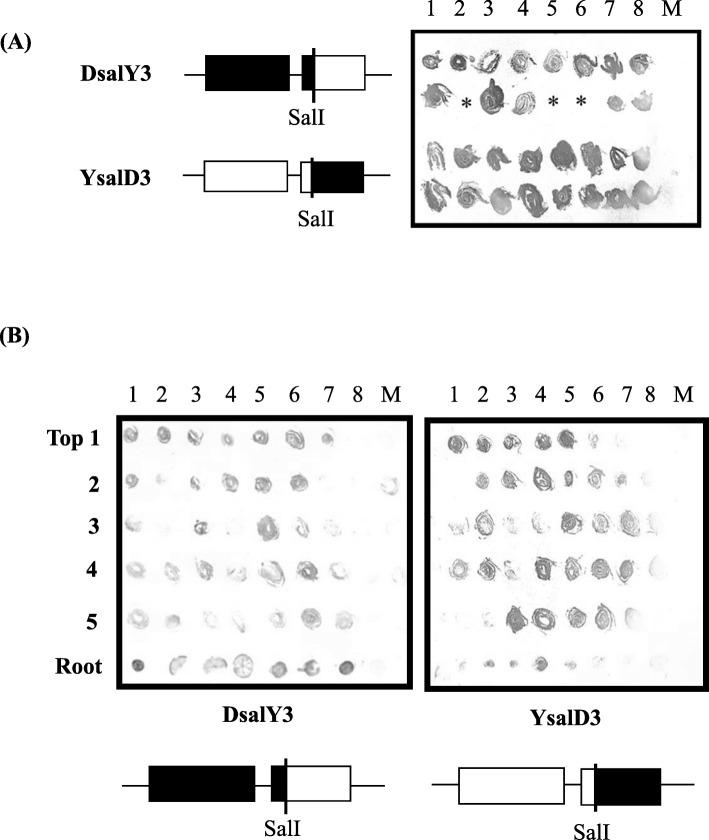
Accumulation of CMV in radish (cv. Tokinashi). **a** Detection of CMV in inoculated cotyledons at 10 dpi and **b** in upper, non-inoculated leaves and root at 21 dpi by tissue immunoblotting using antiserum against CMV-Y CP. Cotyledons of 7-day-old seedlings were mechanically inoculated with chimeric RNA3s, combined with Y RNA1 and D8 RNA2. Numbers at top represent individual test plants; numbers on the left indicate leaf positions from the top of the plants. M, mock-inoculated plants. Asterisks indicate dead or senesced leaves that could not be analyzed

We next analyzed RNA3 function in viral accumulation and systemic movement. Chimeric RNA3s designated as DsalY3 and YsalD3 were constructed between Y and D8 (Additional file 4: Figure S3). Cotyledons on radish plants were inoculated with the recombinant viruses (Y RNA1, D8 RNA2, and the chimeric RNA3s). Tissue immunoblots showed that the recombinant viruses accumulated not only in the inoculated cotyledons, but also in the upper, non-inoculated leaves and roots of radish plants (Table 3, Fig. 5). The results indicated that viral factors involved in systemic movement are present in both the MP and CP regions that border the SalI site in the N-terminal region of the CP in RNA3 (Additional file 4: Figure S3).

**Table 3:** Chimeric and point-mutated RNA3s analysis in radish by tissue immuno-blotting assay

Inoculum	Inoculated leaves^a^	Upper leaves^b^
Y1 D2 YsalD3	8/8^c^	8/8^c^
Y1 D2 DsalY3	8/8	7/8
Y1 D2 Y3MP51NS	8/8	8/8
Y1 D2 Y3CP17LP	8/8	8/8
Y1 D2 Y3CP129SP	8/8	8/8
Y1 D2 Y3CP17LP129SP	8/8	8/8
Y1 D2 Y3MP51NSCP17LP	8/8	8/8
Y1 D2 Y3MP51NSCP129SP	8/8	8/8
Y1 D2 Y3MP51NSCP17LP129SP	8/8	8/8

Data were recorded at ^a^10 dpi; ^b^21 dpi; ^c^Number of infected plants/number of inoculated plants. Some samples with very low levels of viral accumulation were included as positive

**Fig. 6:**
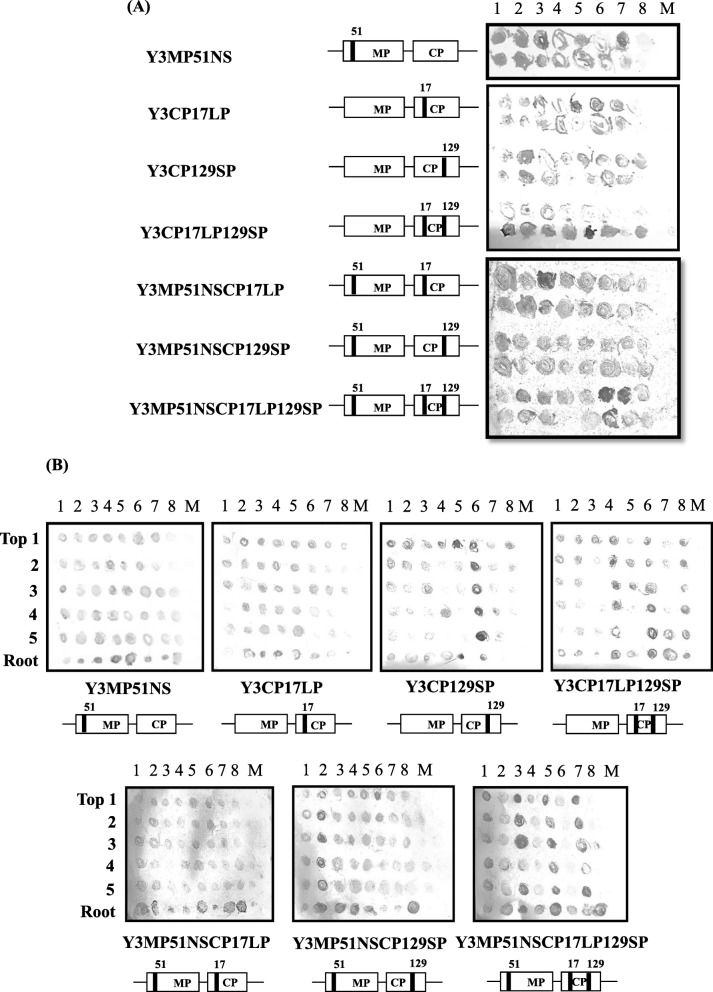
Accumulation of CMV in radish (cv. Tokinashi). **a** Detection of CMV in inoculated cotyledons at 10 dpi and **b** in upper, non-inoculated leaves and root at 21 dpi by tissue immunoblotting using antiserum against CMV-Y CP. Cotyledons of 7-day-old seedlings were mechanically inoculated with point-mutated RNA3s in the MP and CP or both, combined with Y RNA1 and D8 RNA2. Numbers at top represent individual test plants; numbers on left indicate te leaf positions from the top of the plants. M, mock-inoculated plants. Asterisks indicate dead or senesced leaves that could not be analyzed

To further verify differences in the amino acid sequences of the MP and CP by analyzing amino acid similarity among several CMV strains (Additional file 7: Figures S5 and S6), we examined point-mutated RNA3s combined with Y RNA1 and D8 RNA2 using in vitro transcripts (Table 3, Fig. 6). We first analyzed the function of the amino acid position 51 of MP in systemic viral infection. In plants inoculated with Y1D2Y3MP51NS, the virus spread into the upper, non-inoculated leaves (Fig. 6b), suggesting that Ser^51^ in the MP of CMV-D8 plays an important role in systemic infection in radish. We then examined CP function using recombinant RNA3s which contained a point-mutated CP gene (Y3CP17LP, Y3CP129SP, and Y3CP17LP129SP) (Fig. 6). According to a previous study on strains Y, D8 and KM, the virulence of KM in radish was intermediate compared with that of D8 and Y [35]. Additionally, strain KM could infect radish systemically, not strain Y even though their amino acids differ at only positions 17 and 129 in the CP compared with Y CP (Additional file 7: Figure S6), suggesting that amino acids 17 and 129 are involved in systemic infection. Thus, we selected these two amino acids to determine their functions.

**Fig. 7:**
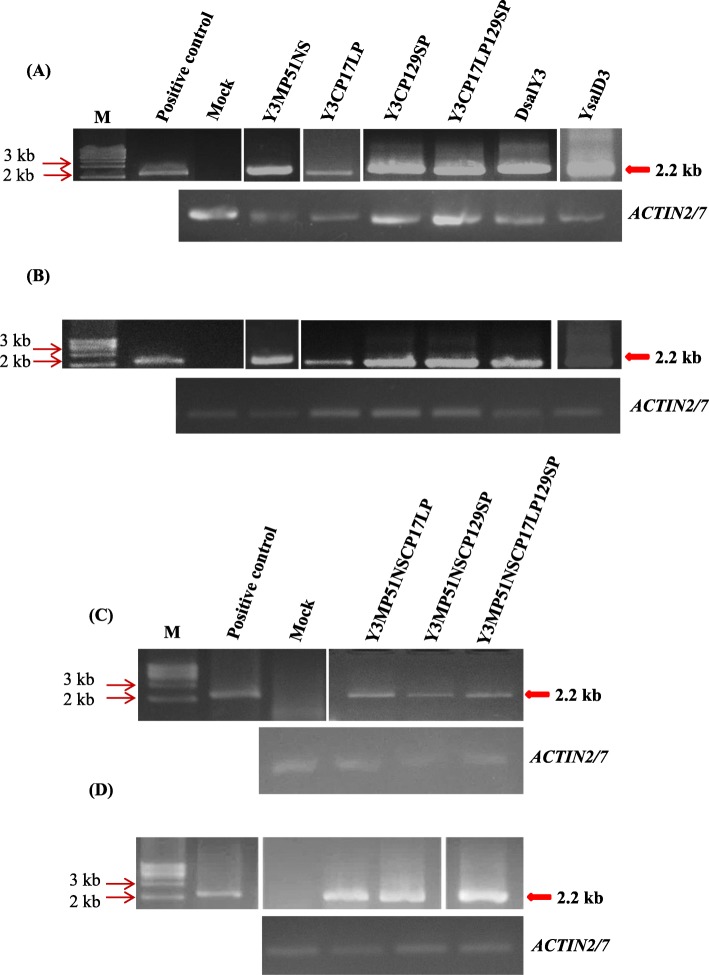
**a**, **c** Detection of CMV in the inoculated cotyledons at 10 dpi, and **b**, **d** in the upper, non-inoculated leaves at 21 dpi by RT-PCR targeting RNA3. Total RNAs were extracted from the plants inoculated with chimeric or point-mutated RNA3s, combined with Y RNA1 and D8 RNA2

Inoculation of plants with the point-mutated viruses (Y RNA1, D8 RNA2 and point-mutated RNA3s) demonstrated that both Pro^17^ and Pro^129^ enabled CMV to infect radish systemically. We further confirmed the results from the MP- and CP-mutated viruses using doubly and triply point-mutated RNA3s (Y3MP51NSCP17LP, Y3MP51NSCP129SP, and Y3MP51NSCP17LP129SP) (Fig. 6). All of the mutants infected all of the inoculated plants. All the results from the mutated RNA3s were verified by RT-PCR targeting CMV RNA3 (Fig. 7). We thus proved that the C-terminal region of 2a, entire 2b are indispensably involved in virus systemic movement in radish with supplementary function by either Ser^51^ in 3a or Pro^17^/ Pro ^129^ in CP of CMV-D8. Nucleotide sequence analyses proved that there were no additional mutations or reversions to wild type after infection in the radish plants.

## Discussion

The viral factors in RNA2 and RNA3 of CMV-D8; C-terminal region of 2a, entire 2b, and either Ser^51^ in 3a or Pro^17^/ Pro ^129^ in CP, contribute to systemic infection in radish cv. Tokinashi, agreeing with and extending the results of our previous study on cv. Akidumari that systemic infectivity of D8 in radish is determined by viral factors in RNA 2 and RNA 3 [39]. D8 accumulates to significantly higher levels than Y does in the inoculated leaves, suggesting that a higher rate of replication and/or higher levels of accumulation of the virus are important for systemic infection in radish.

In the present study, Y2(D2a-C/D2b), which includes a 2a/2b-overlapped region of 2a and the entire 2b of D8, was shown to have a crucial role in systemic infection in radish. Accumulation if the mutant with inhibited expression of 2b, D2(D2a-C/D2b stop) was low even in the inoculated cotyledons, and it failed to move to the upper, non-inoculated leaves. Similar results were obtained for plants inoculated with Y2(∆Y2a-C/D2b), D2(D2a-C/∆D2b-C) or Y2(D2b-C). Cillo et al. [5] reported that the entire 2b, which partially overlaps the C-terminal region of 2a, and adjacent RNA2 sequences are involved in pathogenicity of CMV in tomato. Du et al. [8] also revealed that the same region in RNA2 plays an important role in viral RNA accumulation and symptom induction in *Nicotiana glutinosa*. Here, the use of Y2(∆Y2a-C/D2b) and D2(D2a-C/D2b stop) also showed that the C-terminal region of 2a plus the entire 2b are needed for systemic infection of radish by CMV. From a different view, 2b requires the function of the C-terminal region of 2a to contribute to systemic movement in radish. Spread of CMV without the 2b gene is reduced through epidermal cells, leading to a reduced rate of systemic movement that is host-specific [36] and host-age dependent [16]. Taken together, the results suggested that 2a/2b-overlapped region of 2a and entire 2b of D8 are required for systemic infection and/or to counteract unidentified defensive responses in radish.

Involvement of the MP and CP of D8 in systemic movement also was revealed by the use of several recombinant mutants of RNA3. The reassortants containing chimeric RNA3s showed that not only the MP but also the CP of CMV-D8 possess the ability to guide the long-distance movement of the virus. Takeshita et al. [40] reported that the amino acid at position 51 in the MP and 129 in the CP are primary and secondary determinants, respectively, in systemic infectivity and in the induction of severe chronic symptoms in bottle gourd. Unlike the case in radish, Asn^51^ in MP from CMV-Y, but not Ser^51^ in MP from CMV-D8, facilitated systemic infection in bottle gourd. Two amino acid positions (Lys^51^ and Phe^240^ in the MP) from CMV-Sny were responsible for the restricted movement in cucurbit hosts [17], but increased accumulation of the MP up to 50-fold in tobacco [11]. When all these results are considered, the function of the amino acid at position 51 in the MP probably depends on host species. On the other hand, Pro^129^ in CP in the background of CMV-D8 appears to facilitate systemic infection of CMV in both bottle gourd and radish. In addition, Pro^17^ in CP of CMV-D8 seems to have a minor role in systemic infection of CMV-D8 in radish. In previous studies, CP or the amino acid at position 129 in CP of CMV has been reported as a determinant for systemic infection and symptom phenotypes [19, 25, 26, 32, 38, 40]. Ser^129^, but not Ser^17^, in the CP from CMV-Y induces pin-point necrotic lesions in the inoculated leaves of bottle gourd and chlorotic spots in those of tobacco [38, 40]. Saitoh et al. [32] documented that vascular movement of CMV was regulated by an amino acid alteration at positions 17 (Leu to Pro), 25 (Ser to Pro), 28 (Ser to Ala) and 129 (Ser to Pro) in the CP of CMV-Y in *Cucumis figarei*. In our case, however, serine was found at amino acid positions 25 and 28 of both strains, assuming that amino acid at positions 25 and 28 had no effect on systemic movement, and distribution of the virus was mainly controlled by the amino acid at positions 17 and 129.

Recombination or reassortment of genomic segments between different subgroups has revealed an important function for amino acid changes in host adaptation of CMV virus, which can thereby alter its host range and virulence [7, 29, 31, 39, 40]. A host-range determinant(s) of CMV has been documented in various host species [25, 26, 40] and mostly in CMV RNA3 [13, 29]. We aligned several CMV strains including CMV-Y and CMV-D8 between different subgroups belonging to subgroups IA and IB. Except for CMV-O, which is in subgroup IA, in other CMV strains, Asn is in position 51 in the MP instead of Ser as in D8. All CMV strains except Y have Pro at position 17 in the CP. In addition, amino acid 129 in all CMV strains is also Pro, except in Y and CMV-Nt9. CMV-D8 RNA3 is not clearly different from other strains in the two subgroups at amino acids 51 in the MP and 17 and 129 in the CP (Additional file 7: Figures S5 and S6). Therefore, the difference in RNA3s between subgroups IA and IB does not significantly impact the systemic infectivity of CMV. Rearranged genomic RNA segments of CMV-D8 might reflect natural selection of the most adaptive RNA3 irrespective of the two subgroups.

Hwang et al. [14] used a yeast two-hybrid assay to show that CMV 2a interacts with 3a to play a role in cell-to-cell movement. The determinant of systemic movement of CMV in squash was mapped on 2a and 3a [4]. Additionally, we here report further complex interactions among CMV proteins required for systemic infection in radish.

## Conclusion

The C-terminal region of 2a protein, the entire region of 2b protein, and supplementary function of either Ser^51^ in 3a or Pro^17^/Pro ^129^ in CP facilitate systemic infectivity in radish and provide a clue on the mechanism by which CMV adapted to radish. A histochemical analysis and further studies on the interactions among the viral proteins are needed to dissect the infection dynamics of the mutants and determine the effects of viral factors on vascular spread in radish.

## Supplementary information

**Additional file 1: Figure S1.** (A) Mild systemic mosaic induced by CMV-D8 in radish (cv. Akidumari) [39]. (B) No symptoms were observed on upper, non-inoculated leaves of radish plants (cv. Tokinashi) after inoculation of cotyledons with wild type, pseudorecombinant, or recombinant viruses.

**Additional file 2: Table S1.** List of primer sequences of point-mutated RNA3 constructs.

**Additional file 3: Table S2.** List of primer sequences for recombinant and mutant RNA2s plasmid construction.

**Additional file 4: Figure S2.** Schematic diagram of mutant and recombinant RNA2 constructs. **Figure S3.** Schematic diagram of chimeric and point-mutated RNA3 constructs.

**Additional file 5: Table S3.** List of primer sequences for RT-PCR and relative RT-qPCR.

**Additional file 6: Figure S4.** Alignment of amino acid (aa) sequences of the 2a protein (A) and the 2b protein (B) between CMV-D8 and CMV-Y. Identical nucleotides are indicated by a period (.).

**Additional file 7: Figure S5.** Alignment of amino acid sequences of MPs between subgroup IA and IB of CMV. Red rectangle indicates different amino acids at position 51. **Figure S6.** Alignment of amino acid sequences of CPs between subgroup IA and IB of CMV. Red rectangles indicate different amino acids at position 17 and 129.

## Data Availability

All data supporting the conclusions of this article are included in this published article.

## Abbreviations

A (Ala): Alanine
BSA: Bovine serum albumin
CMV: Cucumber mosaic virus
CP: Coat protein
D1D2Y3: RNA 1 and 2 from CMV-D8, and RNA 3 from CMV-Y
D1Y2D3: RNA 1 and 3 from CMV-D8, and RNA 2 from CMV-Y
D1Y2Y3: RNA 1 from CMV-D8, and RNA 2 and 3 from CMV-Y
D8: CMV-D8
F (Phe): Phenylalanine
IgG: Immunoglobulin G
K (Lys): Lysine
KM: CMV-KM
L (Leu): Leucine
MP: Movement protein
N (Asn): Asparagine
NaCL: Sodium chloride
ORF: Open reading frame
P (Pro): Proline
PBST: Phosphate-buffered saline with Tween 20
RT-PCR: Reverse transcription polymerase chain reaction
RT-qPCR: Quantitative reverse transcription polymerase chain reaction
S (Ser): Serine
Y: CMV-Y
Y1D2D3: RNA1 from CMV-Y, RNA2 and RNA3 from CMV-D8
Y1D2Y3: RNA 1 and 3 from CMV-Y and RNA2 from CMV-D8
Y1Y2D3: RNA 1 and 2 from CMV-Y and RNA3 from CMV-D8
