# Supplementary material for: The 2b protein and C-terminal region of the 2a protein indispensably facilitate systemic movement of cucumber mosaic virus in radish with supplementary function by either the 3a or the coat protein
Source: Virol J. 2020 Apr 7;17:49. doi: 10.1186/s12985-020-01303-3 (PMC7140367; doi:10.1186/s12985-020-01303-3)
Supplement: Supplementary file 1 — Additional file 1: Figure S1. (A) Mild systemic mosaic induced by CMV-D8 in radish (cv. Akidumari) [39]. (B) No symptoms were observed on upper, non-inoculated leaves of radish plants (cv. Tokinashi) after inoculation of cotyledons with wild type, pseudorecombinant, or recombinant viruses. [file 12985_2020_1303_MOESM1_ESM.docx]

**
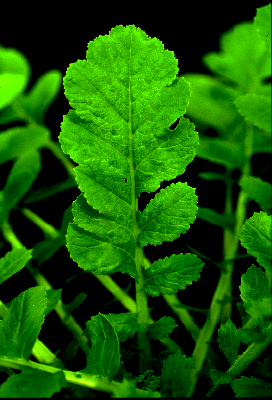
**

**Supplementary Figure S1 (A) Mild systemic mosaic induced by CMV-D8 in radish (cv. Akidumari) (Takeshita; unpublished data).**


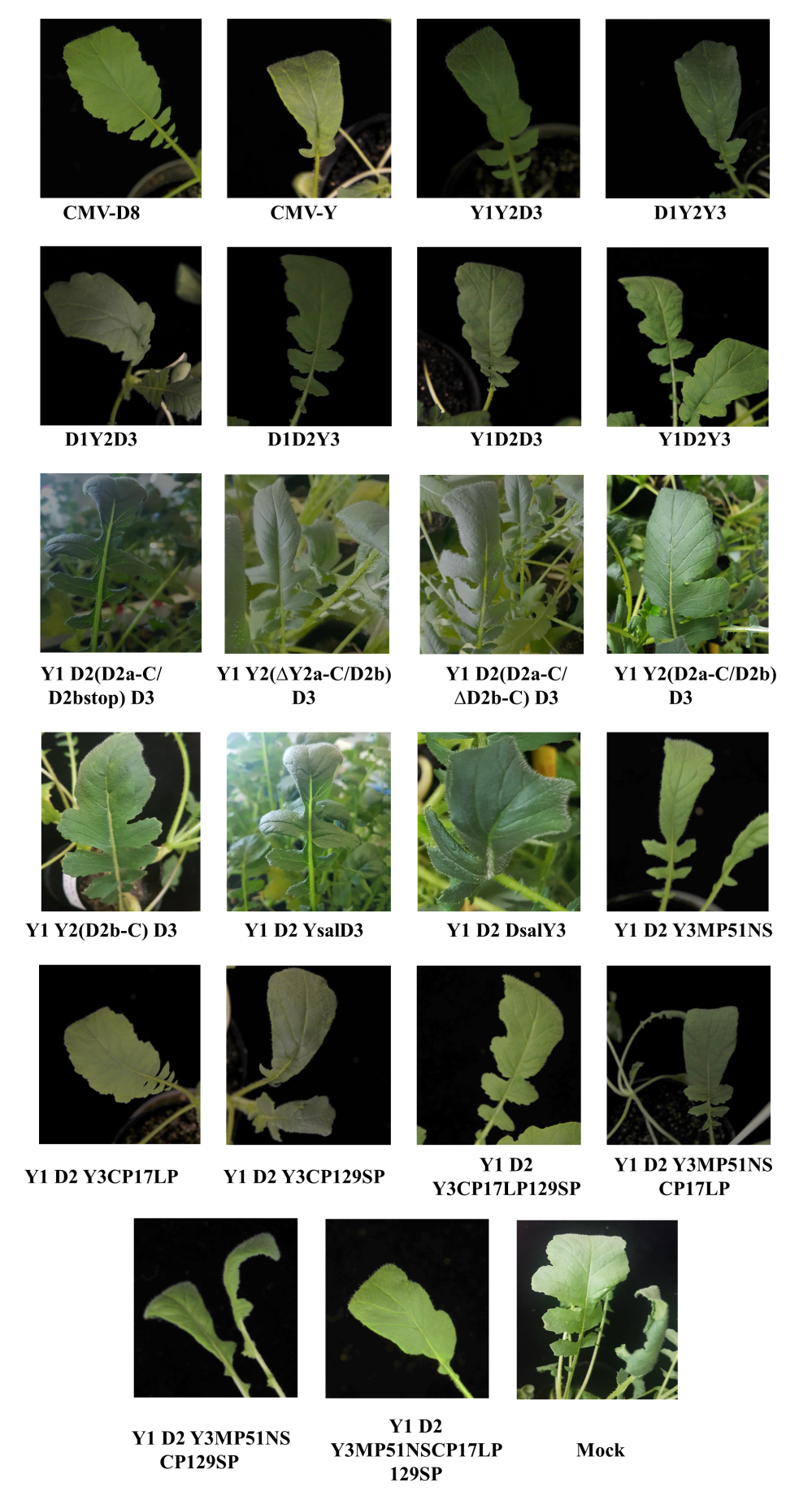


**Supplementary Figure S1 (B)** No symptoms were observed on upper, non-inoculated leaves of radish plants (cv. Tokinashi) after inoculation of cotyledons with wild type, pseudorecombinant, or recombinant viruses.
