# Supplementary material for: The 2b protein and C-terminal region of the 2a protein indispensably facilitate systemic movement of cucumber mosaic virus in radish with supplementary function by either the 3a or the coat protein
Source: Virol J. 2020 Apr 7;17:49. doi: 10.1186/s12985-020-01303-3 (PMC7140367; doi:10.1186/s12985-020-01303-3)
Supplement: Supplementary file 2 — Additional file 2: Table S1. List of primer sequences of point-mutated RNA3 constructs. [file 12985_2020_1303_MOESM2_ESM.docx]

**Supplementary Table S1 List of primer sequences of point-mutated RNA3 constructs**

| **Constructs** | **Positions** | **Base changes** | **Oligonucleotide primers** | **Amino acid substitutions** |
| --- | --- | --- | --- | --- |
| pCY3MP51NS | 272 | T to C | d(GGCCGGACTGAAATAGCACTATCAGCGCGC) | Asn to Ser |
| pCY3CP17LP | 1308, 1309 | AA to GG | d(CCACGACGCGGACGACGTCG) | Leu to Pro |
| pCY3CP129SP | 1644 | T to C | -^a^ | Ser to Pro |
| pCY3CP17LP  129SP | 1305, 1306, 1644 | AA to GG,  T to C | - | Leu to Pro,  Ser to Pro |

^a^ Suzuki et al. 1995
