# Supplementary material for: The 2b protein and C-terminal region of the 2a protein indispensably facilitate systemic movement of cucumber mosaic virus in radish with supplementary function by either the 3a or the coat protein
Source: Virol J. 2020 Apr 7;17:49. doi: 10.1186/s12985-020-01303-3 (PMC7140367; doi:10.1186/s12985-020-01303-3)
Supplement: Supplementary file 3 — Additional file 3: Table S2. List of primer sequences for recombinant and mutant RNA2s plasmid construction. [file 12985_2020_1303_MOESM3_ESM.docx]

**Supplementary Table S2 List of primer sequences for recombinant and mutant RNA2s plasmid construction**

| **RNA2 construct** | **Primer** | **Sequence** |
| --- | --- | --- |
| Y2(D2a-C/D2b) | Fw1 | *TTT GAT GTC AAA AAC AAA CAC TGC CTC GAG* |
|  | Rev1 | *CGA AAG AAA TA T GGA* ATT GAA CGA AGG CGC |
|  | Fw2 | GGA GGT TTC AGA ACG ACC CTT CCG CCC ATT |
|  | Rev2 | AAAAAGCGGCCGCTGGTCTCCTT |
|  | Fw3 | TCA ATT CCA TAT TTC TTT CGC TGT TTA TTG |
|  | Rev3 | AGG GTC GTT CTG AAA CCT CCC CTT CCG CAT |
| Y2(D2b-C) | Fw1 | *TTT GAT GTC AAA AAC AAA CAC TGC CTC GAG* |
|  | Rev1 | *AGC AGA GAC GAA GGT* GCT TCC TAC CAT TCT |
|  | Fw2 | GAC GAT ACA GAT TGG TTC GCT GGT AAC GAA TGG GCG GAA GGG TCG TTC TGA ACC TC |
|  | Rev2 | GAA ATC ATG GTC TTC TTC CGC TGG CAA CGT GAA ACA AGG GGC CTC AGG CTC GGG TG |
|  | Fw3 | GGA GGT TTC AGA ACG ACC CTT CCG CCC ATT |
|  | Rev3 | AAAAAGCGGCCGCTGGTCTCCTT |
| D2(D2a-C/D2b stop) | Fw | **A**GAACGAAGGCGCAATGACAAAC |
|  | Rev | ATTCCATCTTTCTTCTTTCGCTGCTTAGTG |
| D2(D2a-C/  ∆D2b-C) | UP inverse | AAAAAACGCGTGTACGTAAATAGCTGAACCTCCCCTTCCTAATC |
|  | DN inverse | AAAAAACGCGTCAGGCCTCAGACTCGGACAGTCCCACCATGTTC |

The dotted primer sequences indicate primer sequences from CMV-Y; primer sequences in italics are complementary to corresponding RNAs. Bolded nucleotides indicate codons that were replaced with stop codons.
