# Supplementary material for: The 2b protein and C-terminal region of the 2a protein indispensably facilitate systemic movement of cucumber mosaic virus in radish with supplementary function by either the 3a or the coat protein
Source: Virol J. 2020 Apr 7;17:49. doi: 10.1186/s12985-020-01303-3 (PMC7140367; doi:10.1186/s12985-020-01303-3)
Supplement: Supplementary file 4 — Additional file 4: Figure S2. Schematic diagram of mutant and recombinant RNA2 constructs. Figure S3. Schematic diagram of chimeric and point-mutated RNA3 constructs. [file 12985_2020_1303_MOESM4_ESM.docx]

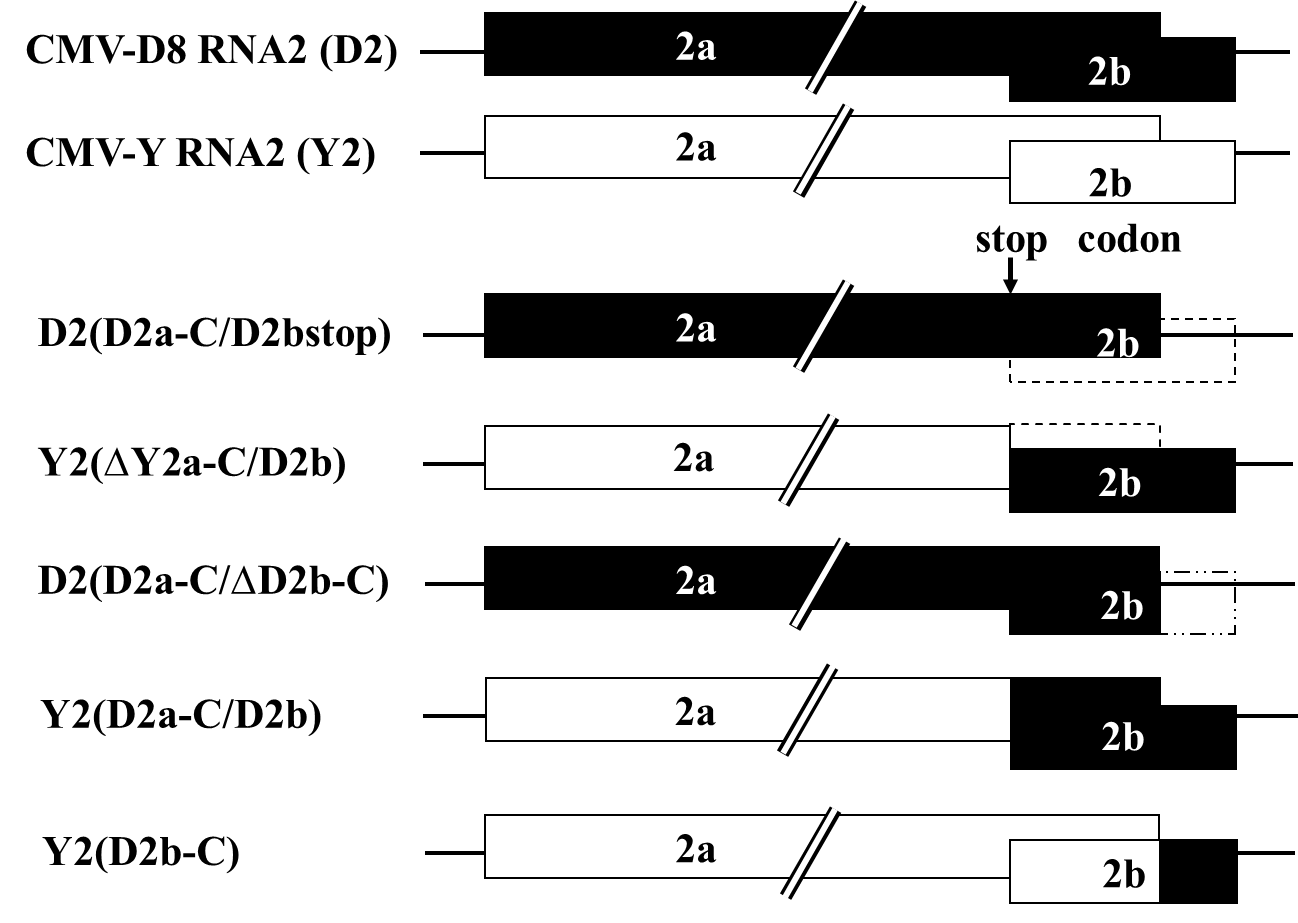


**Supplementary Figure S2 Schematic diagram of mutant and recombinant RNA2 constructs.** D2(D2a-C/D2b stop) mutant was created by introducing a stop codon instead of start codon in the 2b ORF, inhibiting expression of the 2b protein. Y2(∆Y2a-C/D2b) was constructed by inserting the CMV-D8 2b ORF into the C2H1 plasmid vector based on CMV-Y RNA2. D2(D2a-C/∆D2b-C) means deletion of the C-terminal region of the 2b ORF which does not overlap the 2a protein. The recombinant Y2(D2a-C/D2b) was constructed by replacing the 2a/2b-overlapped region of 2a and full region of 2b of CMV-Y RNA2 with the corresponding region of CMV-D8 RNA2. For Y2(D2b-C), only the C-terminal region of CMV-Y 2b was replaced by the corresponding region of CMV-D8 2b. The dotted lines mark the coding region that cannot be expressed. The dashed line marks the deleted coding region.


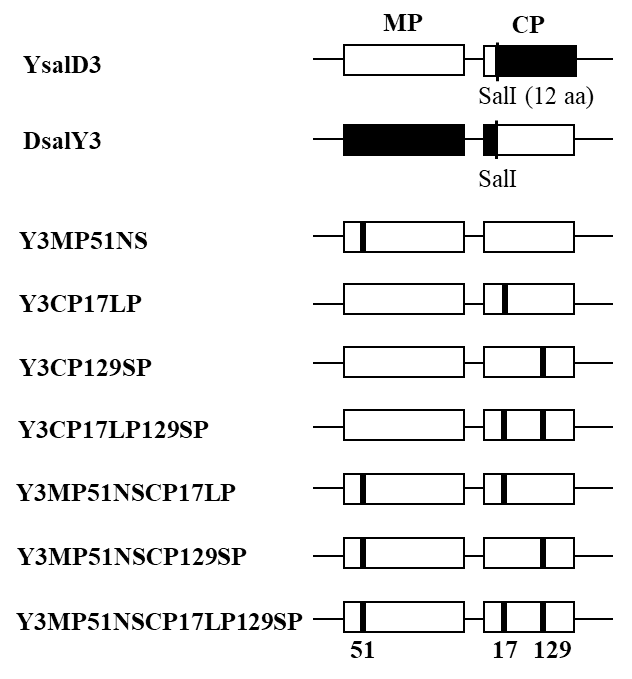


**Supplementary Figure S3 Schematic diagram of chimeric and point-mutated RNA3 constructs.** Chimeric RNA3s between CMV-Y (white boxes) and CMV-D8 (black boxes) were constructed using the SalI restriction site, located at nucleotide 38, which corresponds to amino acid 12, in the CP ORF. Point-mutated RNA3s were created by site-directed mutagenesis of nucleotides encoding amino acids at position 51 in the MP and position 17 and 129 in the CP of CMV-Y by replacing the amino acids from the corresponding positions of CMV-D8.
