# Supplementary material for: The 2b protein and C-terminal region of the 2a protein indispensably facilitate systemic movement of cucumber mosaic virus in radish with supplementary function by either the 3a or the coat protein
Source: Virol J. 2020 Apr 7;17:49. doi: 10.1186/s12985-020-01303-3 (PMC7140367; doi:10.1186/s12985-020-01303-3)
Supplement: Supplementary file 5 — Additional file 5: Table S3. List of primer sequences for RT-PCR and relative RT-qPCR. [file 12985_2020_1303_MOESM5_ESM.docx]

**Supplementary Table S3 List of primer sequences for RT-PCR and relative RT-qPCR**

| **Primer** | **Sequence** |
| --- | --- |
| CMV RNA3 Fw | TTT TGG ATC CAA TTA ATA CGA CTC ACT ATA *GTA ATC TAA CCA CCT GTG TGT* |
| CMV RNA3 Rev | TTT GGC GGC CGC TGG TCT CCT TTT GGA G |
| CMV-D8 RNA2 Fw | *CTT ACT CTC TGA CGA GTT CGG TAA CAC* |
| CMV-D8 RNA2 Rev | CAA TCG ATC AAC AAA GCT CAT GAA GTG |
| ACTIN2/7 Fw | GCATCACACTTTCTACAAC |
| ACTIN2/7 Rev | CCTGGATAGCAACATACAT |
| GAPDH Fw | GAAATCAAGAAGGCTATCAAGGAG |
| GAPDH Rev | TTGTCACCAACGAAGTCAGT |
