# Supplementary material for: The 2b protein and C-terminal region of the 2a protein indispensably facilitate systemic movement of cucumber mosaic virus in radish with supplementary function by either the 3a or the coat protein
Source: Virol J. 2020 Apr 7;17:49. doi: 10.1186/s12985-020-01303-3 (PMC7140367; doi:10.1186/s12985-020-01303-3)
Supplement: Supplementary file 6 — Additional file 6: Figure S4. Alignment of amino acid (aa) sequences of the 2a protein (A) and the 2b protein (B) between CMV-D8 and CMV-Y. Identical nucleotides are indicated by a period (.). [file 12985_2020_1303_MOESM6_ESM.docx]

**(A)**

**(B)**

**Supplementary Figure S4 Alignment of amino acid (aa) sequences of the 2a protein (A) and the 2b protein (B) between CMV-D8 and CMV-Y. Identical nucleotides are indicated by a period (.).**
