# Supplementary material for: The 2b protein and C-terminal region of the 2a protein indispensably facilitate systemic movement of cucumber mosaic virus in radish with supplementary function by either the 3a or the coat protein
Source: Virol J. 2020 Apr 7;17:49. doi: 10.1186/s12985-020-01303-3 (PMC7140367; doi:10.1186/s12985-020-01303-3)
Supplement: Supplementary file 7 — Additional file 7: Figure S5. Alignment of amino acid sequences of MPs between subgroup IA and IB of CMV. Red rectangle indicates different amino acids at position 51. Figure S6. Alignment of amino acid sequences of CPs between subgroup IA and IB of CMV. Red rectangles indicate different amino acids at position 17 and 129. [file 12985_2020_1303_MOESM7_ESM.docx]

**Supplementary Figure S5 Alignment of amino acid sequences of MPs between subgroup IA and IB of CMV. Red rectangle indicates different amino acids at position 51.**

**Supplementary Figure S6 Alignment of amino acid sequences of CPs between subgroup IA and IB of CMV. Red rectangles indicate different amino acids at position 17 and 129.**
